# Gold-standard ontology-based anatomical annotation in the CRAFT Corpus

**DOI:** 10.1093/database/bax087

**Published:** 2017-12-27

**Authors:** Michael Bada, Nicole Vasilevsky, William A Baumgartner, Melissa Haendel, Lawrence E Hunter

**Affiliations:** 1 School of Medicine, Department of Pharmacology, University of Colorado Anschutz Medical Campus, 12801 E. 17th Ave., P.O. Box 6511, MS 8303, Aurora, CO 80045-0511, USA; 2 Ontology Development Group, Library, Oregon Health & Science University, 318 SW Sam Jackson, Park Road, Portland, OR 97239, USA

## Abstract

Gold-standard annotated corpora have become important resources for the training and testing of natural-language-processing (NLP) systems designed to support biocuration efforts, and ontologies are increasingly used to facilitate curational consistency and semantic integration across disparate resources. Bringing together the respective power of these, the Colorado Richly Annotated Full-Text (CRAFT) Corpus, a collection of full-length, open-access biomedical journal articles with extensive manually created syntactic, formatting and semantic markup, was previously created and released. This initial public release has already been used in multiple projects to drive development of systems focused on a variety of biocuration, search, visualization, and semantic and syntactic NLP tasks. Building on its demonstrated utility, we have expanded the CRAFT Corpus with a large set of manually created semantic annotations relying on Uberon, an ontology representing anatomical entities and life-cycle stages of multicellular organisms across species as well as types of multicellular organisms defined in terms of life-cycle stage and sexual characteristics. This newly created set of annotations, which has been added for v2.1 of the corpus, is by far the largest publicly available collection of gold-standard anatomical markup and is the first large-scale effort at manual markup of biomedical text relying on the entirety of an anatomical terminology, as opposed to annotation with a small number of high-level anatomical categories, as performed in previous corpora. In addition to presenting and discussing this newly available resource, we apply it to provide a performance baseline for the automatic annotation of anatomical concepts in biomedical text using a prominent concept recognition system. The full corpus, released with a CC BY 3.0 license, may be downloaded from http://bionlp-corpora.sourceforge.net/CRAFT/index.shtml.

**Database URL**: http://bionlp-corpora.sourceforge.net/CRAFT/index.shtml

## Background

With the ever-rising amount of biomedical literature, it is increasingly difficult for scientists to keep up with the published work in their fields of research, much less related ones. With the digitalization of much of the literature, natural-language processing (NLP) and mining of publications have become increasingly important in biomedical research and curation (1–5). So too have biomedical ontologies, whose use facilitates curational consistency and furthers semantic integration across disparate resources, and millions of biomedical entities have been annotated with them (6, 7). Particularly relevant to biomedicine are the Open Biomedical Ontologies (OBOs), a set of open, orthogonal, interoperable ontologies formally representing knowledge over a wide range of biology, medicine and related disciplines (8).

Manually annotated document corpora are critical gold-standard resources for the training and testing of biomedical NLP systems (9, 10). This was the motivation for the creation of the Colorado Richly Annotated Full-Text (CRAFT) Corpus, a collection of 97 full-length, open-access journal articles from the biomedical literature (11, 12). Within these articles, each mention of the concepts explicitly represented in eight widely used OBOs has been annotated, resulting in gold-standard ontology-based markup of genes and gene products, chemicals and molecular entities, biomacromolecular sequence features, cells, cellular and extracellular components and locations, organisms, biological processes and molecular functionalities. With the >87 000 concept annotations among the approximately 800 000 words in the 67 articles of the 1.0 release, it is one of the largest gold-standard biomedical annotated corpora. (In the journal article in which we first presented the concept annotations of the CRAFT Corpus [11], we assert a total of approximately 100 000 concept annotations in the v1.0 public release; this includes the approximately 12 000 annotations using Entrez Gene database identifiers, which we do not regard as consistent as the annotations using the classes of the eight OBOs, and which we therefore have recommended not to employ for concept recognition work. The assertion in this paper of a total of approximately 87 000 concept annotations in the v1.0 public release excludes these Entrez Gene annotations, which will not be included in the 2.1 version and subsequence releases; however, they will remain available in the archived 1.0 and 2.0 versions.) In addition to this substantial conceptual markup, the corpus is fully annotated along a number of syntactic and other axes, notably by sentence segmentation, tokenization, part-of-speech tagging, syntactic structure markup, text formatting and document sectioning. This initial release of the CRAFT Corpus has enabled the first comprehensive gold-standard evaluation of prominent concept-recognition systems (13), and it has already been used in multiple projects to drive development of systems for a variety of syntactic and semantic NLP tasks including lemmatization (14), coordination resolution (15) and concept recognition and mapping (16–21). It has additionally been used in the development of more expansive systems focused on tasks such as curation (22, 23), information extraction and discovery (24, 25), function prediction (26), querying and search (27), summarization (28) and visualization (17).

Motivated by the considerable recent interest in the automatic identification of anatomical entities in text and, beyond that, extraction and curation of assertions involving anatomical entities (29–37), we have expanded the semantic markup of the CRAFT Corpus with a large set of manually created concept annotations using the classes of the Uberon ontology, a widely used OBO centered on the representation of anatomical entities and life-cycle stages of multicellular organisms as well as multicellular organisms defined in terms of life-cycle stage and sexual characteristics (38). This newly created set of over 16 000 anatomical annotations in the public release, which has been added for v2.1 of the corpus, is by far the largest publicly available collection of gold-standard anatomical markup and is the first large-scale effort of which we know to manually mark up biomedical text that relies on the entirety of an anatomical terminology, as opposed to annotation with a small number of high-level anatomical categories, as performed in previous corpora. In addition to presenting and discussing this newly available resource, we apply it to provide performance baselines for the automatic annotation of anatomical concepts in biomedical text using a prominent concept recognition system.

## Methods

The OBO-format (http://www.geneontology.org/faq/what-obo-file-format) version of the 2015/04/23 version of the Uberon ontology (i.e. with a specified data version of uberon/releases/2015-04-23/basic.owl in the .obo file), which was the version current at the time of the start of the markup of the CRAFT Corpus with this ontology, was downloaded and a Protégé-Frames (39) ontology project was programmatically created by parsing the .obo ontology file and making use of the Protégé-Frames Java API. Even though Uberon continued to be released in subsequent versions, the aforementioned starting version of Uberon was used throughout the annotation process so as to be consistent (as was done for the previous annotation passes with the ontologies used to create v1.0 of the corpus). As annotation was to be performed in Knowtator [a tab plugin to Protégé-Frames designed to enable markup of text using an ontology as the annotation schema (40)], a Knowtator project was created by manually configuring the generated Protégé-Frames project.

All of the Uberon annotation work was manually performed in Knowtator, with no automatically generated pre-annotation, and the original concept annotation guidelines (41), with minor modifications, were employed throughout. The articles marked up in this work were the same ones marked up for v1.0 of the corpus, each of which was originally selected based on (1) its use as an evidential source for one or more mouse genes/gene products in the Mouse Genome Database to one or more classes from the Gene Ontology and/or Mammalian Phenotype Ontology, and (2) for its unrestrictive licensing terms, i.e. availability in PubMed Central in the form of Open Access XML. Apart from these criteria, the articles cover a wide range of research disciplines, including genetics, biochemistry and molecular biology, cell and developmental biology and even bioinformatics/computational biology.

These articles were annotated in batches of four, except for the last batch of five articles. There was no practicing of text annotation with the Uberon ontology; rather, the primary annotator (NV) started the annotation task with the first batch of articles of the corpus. For the majority of the article batches, markup was created by the primary annotator followed by review of this markup by the annotation lead (MB); for stricter evaluation, the last three article batches (of 4, 4 and 5 articles) were independently marked up by the primary annotator and the annotation lead. The primary annotator and annotation lead met by phone after each article batch review (and before continuing onto the next batch) to discuss disagreements in markup noted by the annotation lead and to make edits to the markup where needed. Edits of the markup in previously annotated articles were made to maintain consistency as needed, as concluded in the periodic meetings. Creation or modification of extension classes and markup created with them were performed as annotation issues arose throughout the course of the project. For each article batch, interannotator agreement (IAA) was calculated between either (a) the markup created by the primary annotator and the edited markup resulting from the review by the annotation lead (for the first 21 article batches) or (b) the markup independently created by the primary annotator and the annotation lead (for the last three article batches), with a goal of F_1_-scores ≥ 0.9. All IAA statistics were calculated using Knowtator’s built-in functionality.

The UBERON, UBERON+extensions and UBERON+nested Knowtator projects (corresponding to the variant Uberon-based annotation sets) were programmatically created by filtering the appropriate annotations from the UBERON+extensions+nested Knowtator project (which was the project on which the primary annotation efforts were made) via the Protégé-Frames and Knowtator Java APIs. From these projects, the annotation sets were saved as Knowtator XML files, and GENIA Project Markup Language (GPML) and Resource Description Framework (RDF) format versions of the annotation sets were programmatically generated from the latter.

## Results and discussion

Motivated by the considerable recent interest in the automatic identification of anatomical entities in text as well as extraction and curation of assertions involving anatomical entities, we have expanded the semantic markup of the CRAFT Corpus with a large set of manually created concept annotations using the classes of the Uberon anatomical ontology. We are releasing this markup in four sets, differentiated along two axes, resulting in one complete set of annotations and three containing subsets of the complete set. This modularity allows the user to select and work with a preferred annotation set based upon these two differentiating axes.

One axis of differentiation among these annotation sets is the inclusion or exclusion of nested annotations, i.e. annotations of text spans wholly within the text spans of other annotations. For example, the text ‘embryonic tissue’ is annotated with UBERON:‘embryonic tissue’ (UBERON:0005291), and in the annotation sets including nested annotations, there is an additional annotation of the nested ‘embryonic’ with UBERON:embryo (UBERON:0000922). We have created these nested annotations because we believed some users may be interested in recognizing such nested anatomical concepts; furthermore, in preliminary work we have found that some of them will be needed for a future stage of the CRAFT semantic annotation work in which the named concept annotations will be compositionally joined, including relational linkage. At this stage, we have primarily only created nested annotations whose central words are different from the central words of their nesting annotations; thus, there is no additional annotation of the nested ‘tissue’ with UBERON:tissue (UBERON:0000479), as the nesting ‘embryonic tissue’ annotation is already centered on the nested ‘tissue’; however, we may decide to also universally annotate this type of nested annotation in the future. (We have created a very small number of specific instances of nested annotations that are centered on the same anchor word as their nesting annotations, as they will similarly be needed for this future compositional annotation work; however, their number is so small that these can be effectively ignored.) Some automatic concept annotation tools and systems have the capability to create annotations nested within other annotations, though at least some of these systems attempt to create all possible nested annotations in this mode, even those centered on the same words as their nesting annotations (e.g. ‘embryonic tissue’ and ‘tissue’). Since we have not created such nested annotations (i.e. those centered on the same words as their nesting annotations), if the automatic concept annotation tool being used cannot create only nested annotations centered on words different from those of their nesting annotations, then the user would to remove such nested annotations in a post-processing step. In such a case, we would instead recommend that the user more straightforwardly employ one of the annotation sets without nested annotations. However, for the user who wishes to employ these nested annotations, they are included in the UBERON_core+nested and UBERON_core+extensions+nested annotation sets. Additional examples of nested and nesting annotations can be inspected in Table 1.

**Table 1.** Examples of sentences (along with their PubMed IDs) with nested and nesting UBERON annotations in the CRAFT Corpus. For the latter are shown the specific text spans annotated, class primary labels, and class IDs. Note that discontinuous annotations (i.e., annotations composed of two or more discontinuous text spans) have been created for the second and third sentences.

Sentence	Nested/Nesting annotations
Glaucoma involves retinal ganglion cell death and optic nerve damage that is often associated with elevated intraocular pressure (IOP) [1–5]. (PMID:11532192)	‘retinal’: UBERON:retina (UBERON:0000966)
	‘retinal ganglion’: UBERON:‘ganglionic layer of retina’ (UBERON:0001792)
(b) close-up surface view of ventricle of a chimeric heart generated by aggregation of two diploid morulae, one hemizygous for the CK6/ECFP (ECFP+) transgene and the other hemizygous for the YC5/EYFP (EYFP+) transgene. (PMID:12079497)	‘heart’: UBERON:heart (UBERON:0000948)
	‘ventricle of … heart’: UBERON:‘cardiac ventricle’ (UBERON:0002082)
To investigate this pattern in more detail, we hybridized a Tbx15 mRNA probe to a series of transverse sections at E12.5 and observed expression in multiple mesenchymal tissues of the head, trunk, and developing limbs (Figure 4A), much of which is consistent with the skull, cervical vertebrae and limb malformations reported for mice carrying the original droopy ear allele. (PMID:14737183)	‘head’: UBERON:head (UBERON:0000033)
	‘mesenchymal tissues of … head’: UBERON:‘head mesenchyme’ (UBERON:0005253)
	‘trunk’: UBERON:trunk (UBERON:0002100)
	‘mesenchymal tissues of … trunk’: UBERON:‘trunk mesenchyme’ (UBERON:0005256)
	‘limbs’: UBERON:limb (UBERON:0002101)
	‘mesenchymal tissues of … limbs’: UBERON:‘limb mesenchyme’ (UBERON:0009749)
The overall organogenesis of lungs was preserved in Dhcr7-/- pups; four right lung lobes and a single left lobe flanking the heart were easily seen on external examination at birth (Figure 2D). (PMID:15005800)	‘right lung’: UBERON:‘right lung’ (UBERON:0002167)
	‘right lung lobes’: UBERON:‘right lung lobe’ (UBERON:0006518)
Fetal cholesterol can either be synthesized endogenously in fetal tissues or accrued from extra-embryonic tissues such as maternal serum, placenta and yolk sac [39]. (PMID:15005800)	‘embryonic’: UBERON:embryo (UBERON:0000922)
	‘extra-embryonic tissues’: UBERON:‘extraembryonic tissue’ (UBERON:0005292)

The second axis of differentiation among these annotation sets is the inclusion or exclusion of extension class annotations, i.e. annotations made with what we are calling extension classes, which we have defined as extensions of the Uberon ontology (and in some cases, as extensions of other ontologies as well). Biomedical ontologies are generally well-suited for tasks for which they have been purposely developed, including curation of biomedical entities in databases as well as formal knowledge representation and reasoning. However, many of these ontologies have not been developed with natural-language text annotation and mining strongly in mind, and they are often not as well-suited for these tasks. To make these ontologies more usable for concept annotation of biomedical text, we have created and made use of these extension classes as we encountered issues in annotating certain types of concepts in text. However, it is important to note that extension classes were created and used only if we could create formal logical definitions of existing classes from Uberon and other OBOs for them. With such definitions, these extension classes can be integrated with the OBOs whose classes they rely on.

There were several specific motivations for the creation and use of these extension classes. In some cases, we created extension classes to unify semantically similar concepts that we had difficulty consistently differentiating in text; for example, we created UBERON_EXT:muscle_structure_or_tissue and defined it in terms of the classes UBERON:‘muscle structure’ (UBERON:0005090) and UBERON:‘muscle tissue’ (UBERON:0002385) (neither of which subsumes the other), as we were finding it difficult to consistently annotate mentions of ‘muscle’, ‘muscular’, etc. with these two classes. In other cases, we unified semantically exact or similar classes so as to avoid creating multiple concept annotations over the same text spans among our annotation projects; for example, we created GO_UBERON_EXT:basement_membrane [as an extension of both Uberon and the Gene Ontology (42)], defined it in terms of the classes UBERON:‘basement membrane of epithelium’ (UBERON:0005769) and GO:‘basement membrane’ (GO:0005604), and used it to annotate mentions of basement membranes in both this Uberon project and for updated markup previously created with the Gene Ontology Cellular Components subontology (in which GO:0005604 is located). In some other cases, we sought to annotate text (that could not be annotated with existing Uberon classes) with an extension class that could be defined in terms of existing Uberon classes (and in some cases of classes of other ontologies as well); for example, to mark up mentions of ‘craniofacial’, we created and used UBERON_EXT:face_or_skull, defined in terms of UBERON:face (UBERON:0001456) and UBERON:skull (UBERON:0003129), as there was no UBERON class encapsulating both of these. (Some of the extension classes had several motivations, as they are not mutually exclusive.) It must be stressed that we have not created these classes with the intention of advocating their formal acceptance into Uberon (and/or other OBOs on which they are based), particularly as most of them are defined as unions of existing named classes, which, though logically sound, are often not considered ‘natural kinds’ of entities that some in the OBO community believe that ontologies should be generally limited to (43). Rather, they are extensions that have been created in a semantically coherent way with the OBOs expressly to mark up text that otherwise would be difficult to consistently mark up due to the semantic content of the text being ambiguous and/or modestly out of scope of the OBOs on which they are based. For the user who wishes to employ the extension class annotations, they are included in the UBERON_core+extensions and UBERON_core+extensions+nested annotation sets. Specific examples of extension class annotations can be inspected in Table 2.

**Table 2.** Examples of sentences (along with their PubMed IDs) with UBERON extension class annotations in the CRAFT Corpus. For the latter are shown the specific text spans annotated and extension class names.

Sentence	Extension class annotations
The C57BL/6J and 129P3/J groups consisted of approximately equal numbers of males and females. (PMID:11532192)	‘males’: PATO_UBERON_EXT:male_or_bearer_of_maleness
	‘females’: PATO_UBERON_EXT:female_or_bearer_of_femaleness
These observations demonstrate that the pigmentary and craniofacial characteristics of deH are caused by loss of function for Tbx15. (PMID:14737183)	‘craniofacial’: UBERON_EXT:face_or_skull
Interestingly, the level of endogenous muscle PPARδ protein in the transgenic mice was much higher than in the control littermates. (PMID:15328533)	‘muscle’: UBERON_EXT:muscle_structure_or_tissue
Here, we use regulatory information from the mouse Gdf5 gene (a bone morphogenetic protein [BMP] family member) to develop new mouse lines that can be used to either activate or inactivate genes specifically in developing joints. (PMID:15492776)	‘bone’: UBERON_EXT:bone_element_or_tissue
The undulations were accompanied by partial dissolution of the underlying basement membrane (Figure 3K and L). (PMID:15630473)	‘basement membrane’: GO_UBERON_EXT:basement_membrane

Though we recommend making use of these extension classes and their annotations, users who do not wish to work with them can use either the UBERON_core (which contains neither nested nor extension class annotations) or the UBERON_core+nested annotation set. However, even though only small amounts of markup have been created for most of the extension classes, there are considerable numbers of annotations for some of them, which are not otherwise captured (e.g. PATO_UBERON_EXT:female_or_bearer_of_femaleness, PATO_UBERON_EXT:male_or_bearer_of_maleness, UBERON_EXT:bone_element_or_tissue, UBERON_EXT:muscle_structure_or_tissue). We have also included within the distribution a simple text file of mappings of each Uberon extension class to one or more proper Uberon classes semantically closest to it (in that they are the proper Uberon classes with which the corresponding extension classes have been partly defined). Thus, another option for the user who does not wish to make use of these extension classes is to work with one of the annotation sets that include the extension class annotations and use the mappings to replace occurrences of extension class annotations therein with their correspondingly mapped Uberon classes. In those cases, in which an extension class is mapped to more than one Uberon class (e.g. UBERON:muscle_structure_or_tissue), the user will have to decide which class or classes will be used to replace the annotation extension class. Annotations substituted with these mapped Uberon classes are not guaranteed to be semantically correct, but they will at least be semantically close and thus may be acceptable for those users who do not wish to make use of the extension classes and are willing to tolerate a relatively small degree of semantic inexactness.

**Figure 1.**
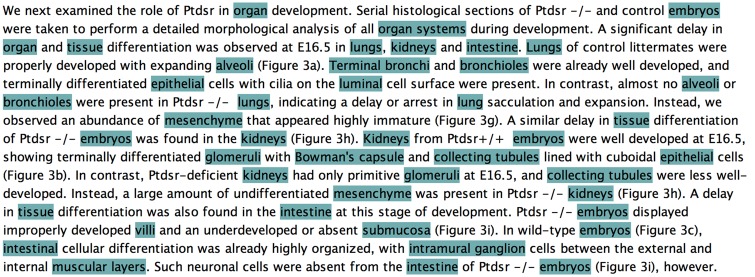
Knowtator screenshot of a paragraph of an article in the public set of the CRAFT Corpus, in which each mention of an anatomical concept explicitly represented in the Uberon ontology has been annotated.

Table 3 presents statistics for the counts of Uberon-based concept annotations, excluding and including nested annotations and those using the newly created extension classes, in the 67 articles constituting the public version of the CRAFT Corpus. (There are an additional 30 articles within the full corpus, which were marked up with >8000 additional annotations; however, as with the previously created concept annotations, the Uberon-based annotations of these articles are temporarily being withheld for use in a future text-mining competition, after which all annotations will be released.) As expected, there are large numbers of Uberon-based named concept annotations created for these articles, ranging from totals of 12 187 to 16 592 annotations in the UBERON_core and UBERON_core+extensions+nested sets, respectively. This translates to averages of between 182 and 248 annotations per article, respectively, and to medians of between 130 and 173 annotations per article, respectively, indicating the presence of articles with large outlying numbers of annotations (as can be seen in the last column of Table 1), which skew the averages up. Figure 1 shows an example paragraph from the CRAFT Corpus in which every mention of an anatomical concept explicitly represented in the Uberon ontology has been annotated.

**Table 3.** Total annotation counts and average, median and maximum counts of annotation counts per article in the four distributed Uberon-based annotation sets of the public version of the CRAFT Corpus

Annotation set	Total # annotations	Average # annotations per article	Median # annotations per article	Maximum # annotations per article
UBERON_core	12 187	182	130	575
UBERON_core+extensions	14 811	221	166	702
UBERON_core+nested	13 625	203	137	739
UBERON_core+extensions+nested	16 592	248	173	811

As a measure of the level of the semantic diversity of anatomical concepts in the corpus, Table 4 presents statistics for the counts of unique concepts employed in the Uberon-based annotations of the public subset of the corpus, again excluding and including nested annotations and those using the newly created extension classes. Paralleling the total counts of annotations, there is a conceptual richness of the Uberon-based markup, ranging from 842 to 915 unique concepts referenced in the UBERON_core and UBERON_core+extensions+nested sets, respectively. This translates to averages of between 31 and 38 unique concepts mentioned per article, respectively, and to medians of between 25 and 31 unique concepts mentioned per article, respectively, analogously indicating the presence of articles with large outlying numbers of mentioned unique concepts, as can be seen in the last column of Table 4.

**Table 4.** Total counts of referenced unique concepts and average, median and maximum counts per article of referenced unique concepts in the four distributed Uberon-based annotation sets of the public version of the CRAFT Corpus

Annotation set	Total # unique concepts	Average # unique concepts per article	Median # unique concepts per article	Maximum # unique concepts per article
UBERON_core	842	31	25	108
UBERON_core+extensions	889	36	30	125
UBERON_core+nested	867	33	25	118
UBERON_core+extensions+nested	915	38	31	136

The 842 unique concepts occurring among the non-nested core Uberon annotations of the CRAFT Corpus constitute only 6.4% of the 13 082 classes of the version of the Uberon ontology used for this annotation project. Such a small percentage may be surprising, but similarly low percentages of ontology classes occurring in text have long been observed. For example, Verspoor *et al.* found that only 6% of Gene Ontology class labels (and only 3.4% of multiword labels) directly occurred in a 2.3-million-word corpus of 9336 PubMed abstracts (44). Similarly, Beisswanger *et al.* found only 15% and 9% of the classes of over 800 000 classes from 80 OBOs in a Medline corpus of 316 250 abstracts and a PubMed Central corpus of 6342 articles, respectively (45). The most straightforward explanation for these low percentages is simply that the large majority of the classes of the Uberon ontology (and other OBOs) represent very specific concepts that seldom appear in text. Some such concepts presumably appear at least in very low numbers in very large corpora such as those examined by Verspoor *et al.* and Beisswanger *et al.* However, in these studies the concepts were searched automatically, with the former only employing exact string matching and the latter additionally only making use of relatively minor lexical and stemming variations; thus, many occurrences of specific concepts appearing as more lexically and/or syntactically distant variations of the class labels and synonyms contained in the ontologies may have been missed. Conversely, though we have employed a much more thorough manual approach, the number of unique concepts annotated remains low due to our public corpus having a much smaller size of 67 articles. It also seems likely that many specific classes are more detectable through the composition of several more atomic concept annotations and/or use of coreferential information, e.g. logically composing a ‘left kidney’ mention and a separate but coreferential ‘interstitium’ mention to form UBERON:‘left kidney interstitium’ (UBERON:0018113). We also note that there were similarly low percentages of unique ontology classes occurring among the annotations of the 1.0 release of the CRAFT Corpus, ranging from 0.04% of all NCBI Taxonomy entries to 18.5% of Cell Ontology classes.

**Figure 2.**
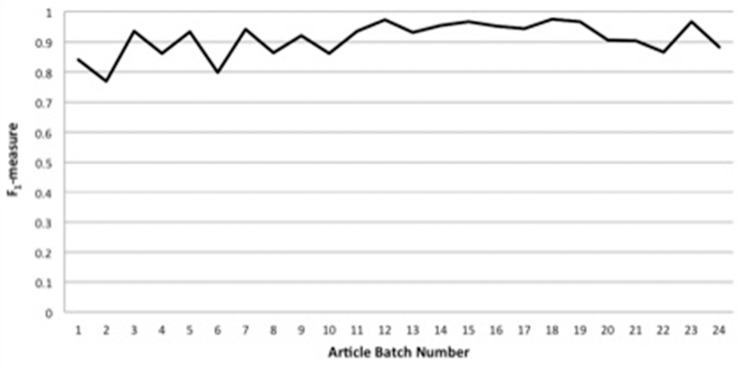
Interannotator agreement statistics between the primary annotator and the annotation lead in the form of F1-measure versus article batch number.

Figure 2 displays IAA statistics between the primary annotator and the annotation lead in the form of F_1_-measure [the harmonic mean of precision and recall (46)] versus article batch number. As can be seen, we have been able to achieve consistently high agreement, with F_1_-measures for article batches of nearly always >0.85, and usually >0.9. As described in the Methods section, most of the concept-annotation evaluation was done in a single-blind fashion, in which the annotation lead reviewed the markup of the primary annotator. However, to demonstrate that this single-blind review did not result in overly generous scoring, the last three article batches (of four, four and five articles) of annotation with the Uberon ontology were annotated in a double-blind fashion, in which neither annotator saw the other’s markup. (We decided to perform the double-blind evaluation on the last three batches of articles so as to avoid conflation of this aspect of the annotation task with the tendency of annotation consistency to be lower toward the beginning of an annotation process due to lower familiarity with the annotation schema and task, and we present the statistics for these last three batches as our highest-achieved double-blind IAA scores.) As can be seen from the last three data points of Figure 2, the F_1_-measure scores for these last three batches, averaging to 0.91, are in line with the scores for the previous batches.

As can be seen in the comparative annotation counts in Tables 3 and 5, this new set of Uberon-based annotations for the CRAFT Corpus is by far the largest collection of gold-standard anatomical markup among publicly available biomedical corpora. These related corpora, including the AnEM (32), CellFinder (31), GENIA (47), MERLOT (48), MiPACQ (49) and MLEE (33) corpora, each contain between 900 and 4500 anatomical annotations. [For the AnEM and MLEE corpora, the annotations for the cell, cellular component and pathological formation categories were not included in the counts shown here, as these categories are outside the scope of the Uberon ontology; furthermore, annotations have already been created and released for mentions of cells and cellular components for v1.0 of the CRAFT Corpus using the Cell Ontology (50) and the Gene Ontology Cellular Components subontology (42), respectively, and we plan to annotate pathological entities separately in the future with a different ontology.] The developers of the Cancer Genetics corpus (37), which also contains some anatomical markup, have reported a total of 21 683 entity annotations in their corpus, but this number also includes annotations of cells, cellular components, chemicals, genes and gene products and protein and DNA domains and regions, so the number of anatomical annotations is presumably much smaller than this reported number. The anatomical markup in the CRAFT Corpus thus constitutes a large and valuable new source of gold-standard data focused on a semantic domain of considerable interest to the biomedical text-mining and curation communities.

**Table 5.** Counts of annotations and semantic categories analogous to those in the Uberon ontology for other gold-standard corpora in which anatomical entities have been marked up

Corpus	# Analogous anatomical annotations	# Analogous anatomical categories
AnEM	1792	8
CellFinder	913	1
GENIA	1167	2
MERLOT	4449	1
MiPACQ	3652	1
MLEE	1346	8

In addition to the amount produced, our newly created anatomical markup can be said to be considerably more semantically rich than related gold-standard annotated biomedical corpora, as can be seen in the comparative numbers of semantic annotation classes/categories used in Tables 4 and 5. The AnEM, Cancer Genetics and MLEE corpora each employed eight high-level anatomical annotation categories, and the remainder only one or two. (For the former, the cell, cellular component and pathological formation categories were again not included, as explained in the previous paragraph.) In contrast, there are totals of between 842 and 915 Uberon and Uberon-based classes used among the four distributed annotation sets of the CRAFT anatomical markup, and averages of 31 to 38 unique Uberon and Uberon-based classes referenced per full-text journal article. This semantic richness can be attributed to our concept annotation guidelines, which dictate that an annotation should in general only be created when the use of an ontology class to annotate selected text will not result in semantic loss of the selected text; that is, the selected text should not be more specific than the ontology class used to annotate it. As a result, many specific anatomical classes are used to annotate the many specific mentions of anatomical concepts that would instead be annotated with high-level anatomical categories in other corpora. This semantic markup specificity is crucial in projects in which large amounts of text—even the entire biomedical literature—are automatically annotated and then mined for knowledge discovery or hypothesis generation and evaluation (51, 52), as voluminous outputs of automatically generated markup cannot possibly be evaluated or analyzed by humans. Additionally, mined knowledge referring only to high-level semantic categories is not likely to be interesting or even relevant to the research question under investigation. Furthermore, the CRAFT anatomical markup can be used in concert with the semantic markup already created for the public version of the corpus, including large amounts of similarly specific markup for genes and gene products, biological sequence features, chemical entities, cells, cellular and extracellular components and locations, organisms, biological processes and molecular functionalities. Together, these semantic annotations can serve as a vital gold-standard resource to train and test computational systems to mark up text for a wide variety of biomedical concepts, which in turn can be utilized for more sophisticated information extraction.

By using the classes of the Uberon ontology to annotate mentions of anatomical concepts in biomedical text, users can additionally benefit from the semantic richness of the ontology itself and its many linkages to other resources. It is an ontology with over 13 000 anatomical classes, which, in addition to being labeled with metadata such as natural-language definitions and synonyms, are arranged into a taxonomic hierarchy and further linked to each other through necessary assertions as well as necessary and sufficient logical definitions, all of which can be employed for reasoning and intelligent querying. Additionally, it has been linked via logical definitions to other widely used OBOs, including the Gene Ontology Biological Process subontology [e.g. formally defining GO:‘neural tube closure’ (GO:0001843) in terms of UBERON:‘neural tube’ (UBERON:0001049)] (53), thus enabling further reasoning across these linked ontologies and transitively to yet other ontologies linked to the latter. Users can also take advantage of the large amounts of curated information either directly or indirectly linked to Uberon classes, including phenotype annotations in the Phenoscape (54) and Monarch (55) databases and gene expression data via BgeeDB (56). Furthermore, also within the ontology are nearly 50 000 mappings of UBERON classes to entries in other ontologies and vocabularies, including the FMA (57), MeSH (58), SNOMED (59) and UMLS (60) terminologies as well as many taxon-specific anatomical OBOs [e.g. Adult Mouse Anatomy Ontology (61), Hymenoptera Anatomy Ontology (62)], so users employing other anatomical terminologies for which mappings from Uberon have been specified can also benefit from the Uberon corpus annotations.

As for the concept annotations provided in the initial 1.0 release of the corpus, we have provided these new sets of Uberon-based annotations in an XML-based format generated by Knowtator and in a format based on the W3C standard RDF, which open the annotations up to the Semantic Web community. Additionally, we have provided a version of the annotation sets in the XML-based GPML (63), which allows the annotations to be easily used in software based on the well-known GENIA corpus. However, it is important to note that because discontinuous annotations (i.e. annotations composed of two or more disconnected text spans) cannot be unambiguously represented in GPML, we have excluded all such annotations from this version; thus, the Uberon-based concept annotations in the GPML format are to be regarded as incomplete.

As a service for researchers who are seeking to automatically identify anatomical concepts in biomedical text, we have applied our new set of anatomical annotations toward a comprehensive evaluation of ConceptMapper, a prominent generic concept recognition tool (64), toward the task of annotating specific spans of biomedical text with Uberon and Uberon-based classes. For this study, we made use of a pipeline we previously developed for a comprehensive evaluation of several prominent concept recognition systems (including ConceptMapper), using the annotations made with eight OBOs as part of the v1.0 release of the CRAFT Corpus as a gold standard (13). (Details of the pipeline are discussed in this previously published evaluation.) As ConceptMapper was seen in general to perform best at least in terms of F_1_-score in that evaluation, we have repeated the testing methodology for ConceptMapper, in which a run was made for each of the 576 combinations of its seven customizable parameters (caseMatch, findAllMatches, orderIndependentLookup, searchStrategy, stemmer, stopWords and synonyms), but instead using the CRAFT Uberon-based concept annotations as a gold standard. [This obviously is not intended to be a comprehensive evaluation of all biomedical concept recognition systems (65, 66); rather, it provides a baseline performance against which other systems may be compared as well as a set of reasonable suggestions for researchers who are seeking to automatically identify anatomical concepts in biomedical text.]

We have performed this comprehensive testing of ConceptMapper using the UBERON_core and UBERON_core+extensions annotation sets, the results of which may be inspected in Tables 6 and 7, respectively. For the maximization of precision, whose results are shown in the first rows of Tables 6 and 7, the optimal ConceptMapper parameter configurations encourage exact matches to class labels and synonyms in an attempt to maximize true positives and minimize false positives, at the expense of increased false negatives and decreased recall. For the maximization of recall, whose results are shown in the second rows of Tables 6 and 7, the optimal ConceptMapper parameter configurations encourage much more unfettered matching (including stemming, ignoring of case and word order and use of all synonyms) in an attempt to maximize true positives and minimize false negatives, at the expense of substantially increased false positives and decreased precision. For the maximization of F_1_-measure, whose results are shown in the third rows of Tables 6 and 7, these two extremes are balanced. The optimal ConceptMapper parameter configurations for the UBERON_core and UBERON_core+extensions annotation sets are almost identical, with only minor variations for the maximization of recall. However, the UBERON_core+extensions annotation set yields slightly better performance, likely due to the class labels and synonyms we have created for the extension classes.

**Table 6.** Parameter settings, true positive counts (TPs), false positive counts (FPs), false negative counts (FNs), precision scores (P), recall scores (R), and F_1_-measure scores (F_1_) for ConceptMapper runs found to produce maximal P, R, and F_1_ scores on the publicly released UBERON_core set of concept annotations. (Each bolded number indicates the maximal score of the parameter that was optimized for the given row.)

Concept mapper parameter settings	TPs	FPs	FNs	P	R	F_1_
caseMatch:CASE_SENSITIVE	5208	2132	6979	**0.71**	0.43	0.53
findAllMatches:NO						
orderIndependentLookup:OFF						
searchStrategy:CONTIGUOUS_MATCH						
stemmer:NONE						
stopWords:NONE						
synonyms:EXACT_ONLY						
caseMatch:CASE_IGNORE	9057	39 389	3130	0.19	**0.74**	0.30
findAllMatches:YES						
orderIndependentLookup:ON						
searchStrategy:SKIP_ANY_MATCH						
stemmer:PORTER						
stopWords:NONE						
synonyms:ALL						
caseMatch:CASE_INSENSITIVE	8102	4325	4085	0.65	0.66	**0.66**
findAllMatches:NO						
orderIndependentLookup:OFF						
searchStrategy:CONTIGUOUS_MATCH						
stemmer:PORTER						
stopWords:NONE						
synonyms:EXACT_ONLY

**Table 7.** Parameter settings, true positive counts (TPs), false positive counts (FPs), false negative counts (FNs), precision scores (P), recall scores (R), and F_1_-measure scores (F_1_) for ConceptMapper runs found to produce maximal P, R, and F_1_ scores on the publicly released UBERON_core + extensions set of concept annotations. (Each bolded number indicates the maximal score of the parameter that was optimized for the given row.)

Concept mapper parameter settings	TPs	FPs	FNs	P	R	F_1_
caseMatch:CASE_SENSITIVE	6960	2389	7851	**0.74**	0.47	0.58
findAllMatches:NO						
orderIndependentLookup:OFF						
searchStrategy:CONTIGUOUS_MATCH						
stemmer:NONE						
stopWords:NONE						
synonyms:EXACT_ONLY						
caseMatch:CASE_IGNORE	11 454	34 708	3357	0.25	**0.77**	0.38
findAllMatches:YES						
orderIndependentLookup:ON						
searchStrategy:SKIP_ANY_MATCH or						
SKIP_ANY_MATCH_ALLOW_OVERLAP						
stemmer:BIOLEMMATIZER						
stopWords:NONE						
synonyms:ALL						
caseMatch:CASE_INSENSITIVE	10 467	4688	4344	0.69	0.71	**0.70**
findAllMatches:NO						
orderIndependentLookup:OFF						
searchStrategy:CONTIGUOUS_MATCH						
stemmer:PORTER						
stopWords:NONE						
synonyms:EXACT_ONLY						

## Concurrent and future work on the CRAFT corpus

In addition to the newly created anatomical markup, we are in the process of updating the semantic annotations created for the initial release of the corpus. More specifically, we are using updated versions of the eight OBOs initially used to again review the articles of the corpus and edit, add or delete concept annotations as needed due to changes, additions or obsoletions of classes of the ontologies.

Analogous to the Uberon markup, included in this update are nested annotations as well as annotations made with extension classes, though both of these types of annotations will analogously be modularly available so that researchers can select which of these annotation sets they are interested in using. In addition to this concept annotation update work, which we expect to complete and publicly release soon, we have done a significant amount of preliminary work and will soon begin formal work on the next stage of semantic annotation of the corpus, which we are calling compositional annotation, in which the concept annotations will be joined together, including via relations, to create progressively more complex biological concepts referred to in the text. Through our continuing annotation work we hope to not only maintain the relevancy of the corpus in light of the evolution of these ontologies with periodic updates of the annotations using new versions of the ontologies but also to extend its utility with new markup with additional relevant ontologies as well as with more semantically complex annotations composed from previously generated concept markup.

## Conclusions

Building on its demonstrated utility, we have presented continuing work on the CRAFT Corpus in the form of a new high-quality set of semantic annotations relying on the Uberon anatomical ontology. This is the largest publicly available collection of gold-standard anatomical markup and is the first large-scale effort at manual markup of biomedical text relying on the entirety of an anatomical terminology. These annotations can be used to train and test systems to recognize mentions of anatomical concepts in text, and, in concert with the prodigious amount of previously created semantic and syntactic markup in the corpus, for automatic extraction of assertions involving these entities. We have also ran the ConceptMapper concept recognition system with every one of its hundreds of parameter setting combinations and reported the configurations that maximize precision, recall and F_1_-measure in the automatic recognition of anatomical entities, using our newly created markup as a gold standard. We intend to continue to develop and extend the CRAFT Corpus so as to progressively increase its utility for biomedical NLP researchers and database curators.

*Conflict of interest*. None declared.
